# Molecular Detection of HIV-1 Subtype B, CRF01_AE, CRF33_01B, and Newly Emerging Recombinant Lineages in Malaysia

**DOI:** 10.4269/ajtmh.14-0681

**Published:** 2015-03-04

**Authors:** Jack Bee Chook, Lai Yee Ong, Yutaka Takebe, Kok Gan Chan, Martin Choo, Adeeba Kamarulzaman, Kok Keng Tee

**Affiliations:** Centre of Excellence for Research in AIDS (CERiA), Department of Medicine, Faculty of Medicine, University of Malaya, Kuala Lumpur, Malaysia; AIDS Research Center, National Institute of Infectious Diseases, Toyama, Shinjuku-ku, Tokyo, Japan; Division of Genetics and Molecular Biology, Institute of Biological Sciences, Faculty of Science, University of Malaya, Kuala Lumpur, Malaysia

## Abstract

A molecular genotyping assay for human immunodeficiency virus type 1 (HIV-1) circulating in Southeast Asia is difficult to design because of the high level of genetic diversity. We developed a multiplex real-time polymerase chain reaction (PCR) assay to detect subtype B, CRF01_AE, CRF33_01B, and three newly described circulating recombinant forms, (CRFs) (CRF53_01B, CRF54_01B, and CRF58_01B). A total of 785 reference genomes were used for subtype-specific primers and TaqMan probes design targeting the *gag*, *pol*, and *env* genes. The performance of this assay was compared and evaluated with direct sequencing and phylogenetic analysis. A total of 180 HIV-infected subjects from Kuala Lumpur, Malaysia were screened and 171 samples were successfully genotyped, in agreement with the phylogenetic data. The HIV-1 genotype distribution was as follows: subtype B (16.7%); CRF01_AE (52.8%); CRF33_01B (24.4%); CRF53_01B (1.1%); CRF54_01B (0.6%); and CRF01_AE/B unique recombinant forms (4.4%). The overall accuracy of the genotyping assay was over 95.0%, in which the sensitivities for subtype B, CRF01_AE, and CRF33_01B detection were 100%, 100%, and 97.7%, respectively. The specificity of genotyping was 100%, inter-subtype specificities were > 95% and the limit of detection of 10^3^ copies/mL for plasma. The newly developed real-time PCR assay offers a rapid and cost-effective alternative for large-scale molecular epidemiological surveillance for HIV-1.

## Introduction

Human immunodeficiency virus type 1 (HIV-1) is classified into three major groups (M, N, and O), in which HIV-1 group M is responsible for global pandemic with nine established HIV-1 subtypes and various circulating recombinant forms (CRFs) (www.hiv.lanl.gov). The Joint United Nations Program on HIV/AIDS (UNAIDS) estimated around 35.3 million people living with HIV-1 globally with 1.6 million deaths caused by acquired immunodeficiency syndrome (AIDS) and 2.3 million of new infections in by the end of 2012. In Southeast Asia, an estimated 3.9 million people are living with HIV-1, with 270,000 new HIV-1 infections and 220,000 AIDS-related deaths reported.^1^

The HIV-1 subtype B and CRF01_AE are the predominant genotypes in Southeast Asia in the past decades.^2^ More recently, HIV-1 CRF33_01B has been steadily expanding in the Asia Pacific region (Malaysia, Singapore, Indonesia, Australia, and Hong Kong^3^–^7^) and has caused infections among various risk groups, including the low-risk populations.^8^ Co-circulation of subtype B, CRF01_AE, and CRF33_01B has also led to the emergence of various recombinant variants.^8^ Recombinant lineages with identical recombination structure that caused epidemic spread are classified as CRFs,^9^ whereas individual viral isolates with a distinct recombination pattern are termed as the unique recombinant form (URF). To date, at least nine novel CRFs have emerged as the result of a recombination between various HIV-1 lineages in Southeast Asia: five in Malaysia,^6^,^10^–^13^ three in Thailand,^14^–^16^ and one in Singapore.^17^ Because of the extensive genetic diversity and broad distribution of CRFs among the HIV-1-infected population in Southeast Asia, the availability of a rapid and multi-target subtyping tool will be of importance for high-throughput detection of classical and novel HIV-1 genotypes circulating in the region.

Continual advances in nucleic acid testing have enabled molecular epidemiological surveillance of rapidly evolving human pathogens such as HIV-1 to be carried out. Real-time polymerase chain reaction (PCR) by TaqMan assay uses fluorescent probes that enables simultaneous identification of targeted gene(s) during PCR amplification. Real-time PCR technology is an appealing alternative to conventional PCR as a result of the combination of excellent sensitivity and specificity, low contamination risk, and ease of performance and speed.^18^ Availability of a multiplex real-time PCR tool provides rapid and accurate identification of HIV-1 genotypes in areas where the HIV-1 prevalence is high, allowing large-scale molecular surveillance to be conducted in the most resource effective manner.^19^–^23^ A subtyping strategy that takes into account the spatial diversity of the virus, if applied to prospective cohorts, can be used to track the movement or diffusion of the common and also atypical HIV-1 lineages over the large geographical vicinity. A multi-region subtyping assay can be used as a timely and powerful “CRF discovery” tool in a region where novel CRFs have been continuously emerging.^22^ In this study, we developed an accurate multiplex real-time PCR assay for rapid detection of classical and recently emerging HIV-1 genotypes in Southeast Asia, and discussed its application in studying clinical and field samples in a setting of high HIV-1 genetic complexity. The method described in this study highlights the importance of active molecular surveillance necessary to understand the spatial and temporal dynamics of HIV-1,^8^ which may in turn inform the public health experts of effective intervention strategies, including vaccine selection and development.

## Materials and Methods

### Sample collection and nucleic acid extraction.

A total of 180 clinical plasma specimens from HIV-1-infected individuals from the University Malaya Medical Center (UMMC), Kuala Lumpur collected between 2008 and 2012 were screened for HIV-1 genotypes. The study was approved by the UMMC Medical Ethics Committee. Standard and multilingual consent forms were used and written consent was obtained from all study participants. The HIV-1 viral RNA was purified from plasma samples using the NucliSENS EasyMAG automated platform (bioMerieux, Durham, NC) following the manufacturer's protocol.

### Primers and probes design.

**Figure 1.**
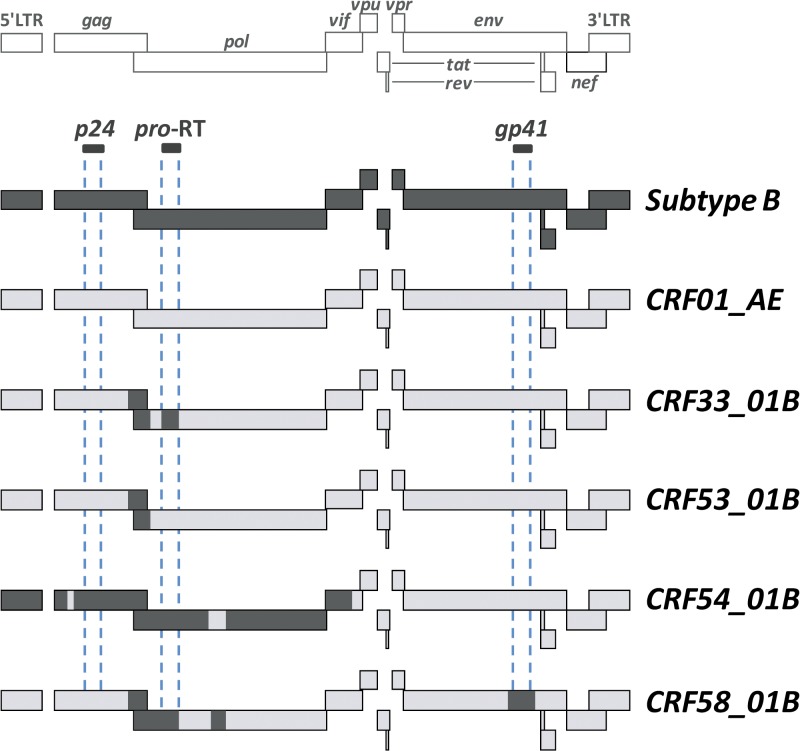
Genome organizations of full-length human immunodeficiency virus type 1 (HIV)-1 subtype B, CRF01_AE, CRF33_01B, CRF53_01B, CRF54_01B, and CRF58_01B. The HIV-1 subtype B and CRF01_AE, shown respectively in dark and light shades, are the putative parental genotypes for the various recombinant lineages (CRF33_01B, CRF53_01B, CRF54_01B, and CRF58_01B) reported previously. Multiplex real-time polymerase chain reaction (PCR) primers and TaqMan probes designed at three different genetic regions (*p24*, *pro*-RT, and *gp41*) are indicated by dashed vertical lines. Real-time PCR results or yield for each genetic region, as indicated by positive or negative amplification, will be used to determine the HIV-1 genotypes.

Complete or near full-length genomes of CRF01_AE (*N* = 596) and subtype B (*N* = 189) of Asian origin were retrieved from the Los Alamos National Laboratory (LANL) HIV sequence database (www.hiv.lanl.gov). The sequences were aligned using the web-based multiple sequence alignment program MAFFT^24^ and manually edited where necessary. Consensus sequences were built and compared for each subtype. Primers and probes for real-time PCR were designed using Primer Express Software v2.0 (Applied Biosystems, Foster City, CA) at three different targeted regions (*gag*, *pol*, and *env* regions) based on the genome organization of HIV-1 subtype B, CRF01_AE, and the mosaic recombinant descendants of CRF33_01B, CRF53_01B, CRF54_01B, and CRF58_01B (Figure 1

). The three genetic regions targeted in this assay were as follows: 1) the *gag p24* region for CRF01_AE and CRF01_AE-origin segments in CRF33_01B, CRF53_01B, and CRF58_01B; 2) the *pol* protease-reverse transcriptase (*pro*-RT) region for subtype B and subtype B-origin segments in CRF33_01B, CRF54_01B, and CRF58_01B; and 3) the *env gp41* region for subtype B and subtype B-origin segment in CRF58_01B. Newly designed primers and probes are listed in Table 1.

### Multiplex real-time PCR.

The HIV-1 RNA was then reverse transcribed into cDNA using SuperScript III RNase H Reverse Transcriptase (Invitrogen, Carlsbad, CA) and random hexamers (Applied Biosystems, Carlsbad, CA). The first round PCR mix contained MyFi Mix, 2× (Bioline, UK), 400 nM of each outer primer, and 3 μL of cDNA. Nuclease-free water was added up to a final volume of 50 μL per reaction. The thermal cycling profile was as follows: initial denaturation at 95°C for 3 min, 30 cycles of 98°C for 20 s, 44°C for 1 min and 72°C for 1 min, and final extension at 72°C for 10 min in a DNA Engine Tetrad2 Thermal Cycler (BioRad, Hercules, CA).

The real-time PCR mix in the second round PCR contained 12.5 μL of SensiFAST Probe Lo-ROX (Bioline), 400 nM of each primer set of inner primer (except 800 nM of forward primer for the *pro*-RT region), 200 nM of probe for *p24* region, 300 nM for *pro*-RT region, and 400 nM for *gp41* region in a final volume of 25 μL. The TaqMan probes were labeled with different fluorescent reporter dyes at 5′-end and each probe selectively hybridized to the specific target region. The probes within the *p24*, *pro*-RT, and *gp41* regions were labeled with the FAM, VIC, and NED dyes (Applied Biosystems), respectively. All probes were labeled with non-fluorescent quencher minor groove binder at the 3′-end. The real-time amplification PCR was performed on the ViiA 7 Real-Time PCR System (Applied Biosystems) with the following thermal profiles: 95°C for 2 min, 40 cycles of 97°C for 15 s, and 55°C for 1 min 30 s. Of note, we have included viral isolates with confirmed HIV-1 genotypes (subtype B, CRF01_AE, and CRF33_01B) previously determined in our laboratory as in-house controls for every run.

A classification system based on the real-time PCR results or yield of the targeted regions were used to interpret the HIV-1 subtype or CRF in the samples (Table 2). A quantification cycle (C_q_) rule^25^ derived based on the targeted regions was applied to improve the accuracy for the differential identification of HIV-1 subtypes/CRFs. The C_q_ value was determined by the lowest take-off point above the background noise of the amplification curve. The optimum difference between the C_q_ values (ΔC_q_) for the *p24*, *pro*-RT, and *gp41* regions of the same sample was determined. The C_q_ values of more than 38 cycles were disregarded. It is also essential to note that both CRF01_AE and CRF53_01B shared similar real-time PCR yield and were not readily distinguished by the assay (Table 2). Genetic sequencing followed by phylogenetic analysis (refer below) were therefore performed for precise genotype classification.

A panel of calibrated standards of HIV-1 subtypes obtained from the NIH AIDS Research and Reference Reagent Program was used for assay validation and the determination of limit of detection (LOD). The panel included HIV-1 subtype B (US1, BK132, and BZ167) and CRF01_AE (CM235, CM240, and IN12), which were originated from the United States, Brazil, Thailand, and Indonesia.^26^ Each sample was run in duplicates. The real-time PCR results were then analyzed and the genotype was determined based on the protocol described previously. A 10-fold serial dilution of standards with known viral load was used to assess the LOD. The HIV-1 negative plasma specimens (*N* = 50) were also used to determine the specificity of the assay.

To validate our method, we tested a different set of field samples collected among fishermen from a rural community in Kuantan (more than 200 km east of Kuala Lumpur) was conducted. A total of 410 subjects with histories of injecting drug use were recruited in 2011. The HIV-1 screening was performed using the ACON HIV Rapid Test Kit (ACON Laboratories, San Diego, CA) and positive results were confirmed with the Intec HIV Rapid Test Kit (Intec Products Inc., Xiamen, China). Plasma samples from HIV-positive subjects were transported to the laboratory in Kuala Lumpur for real-time PCR analysis.

### Sequencing and phylogenetic analysis.

For accurate HIV-1 genotype determination, the conventional direct Sanger sequencing approach and phylogenetic analysis were performed. Nested PCR was performed on each sample using the in-house primers described previously to amplify the *gag*-*pol* gene (HXB2: 625–3440nt).^8^ Sequencing reaction was carried out in an ABI PRISM 3730xl Genetic Analyzer using the BigDye Terminator v3.1 cycle sequencing kit chemistry (Applied Biosystems, Foster City, CA). The nucleotide sequences were edited manually and the *gag-pol* contigs were aligned with the HIV-1 reference subtypes and CRFs downloaded from the LANL HIV sequence database. The reference strains of HIV-1 genotypes used in the alignment were CRF01_AE, subtype B, CRF33_01B, CRF48_01B, CRF51_01B, CRF53_01B, CRF54_01B, and CRF58_01B. Neighbor-joining phylogenetic analysis was carried out using the Kimura 2-parameter model in MEGA version 6.0.^27^ The Recombinant Identification Program (RIP) available at the LANL HIV sequence database and bootscanning analysis were used for the characterization of recombinant mosaic genomes.^28^ The TaqMan real-time PCR assay results were then compared with the phylogenetic data for HIV-1 genotype confirmation.

## Results

### Assay development and validation.

**Figure 2.**
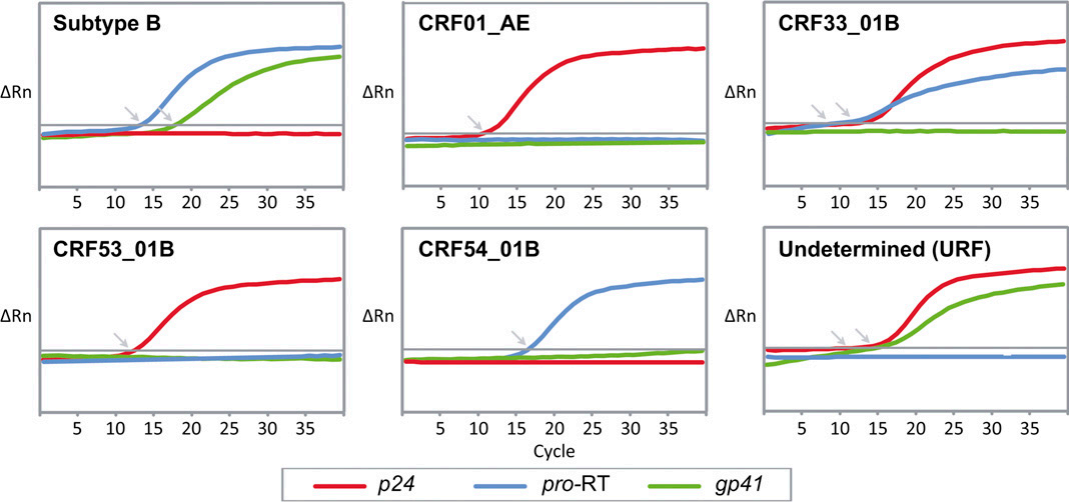
Real-time PCR amplification plot for human immunodeficiency virus type 1 (HIV-1) genotypes. Representative **a**mplification curves of the *p24*, *pro*-RT, and *gp41* genes for subtype B, CRF01_AE, CRF33_01B, CRF53_01B, CRF54_01B, and an undetermined genotype from Kuala Lumpur were shown. The quantification cycle (C_q_) value was indicated by an arrow. CRF01_AE, CRF33_01B, and CRF53_01B showed positive amplification in the *p24* region (red); subtype B, CRF33_01B, and CRF54_01B in the *pro*-RT (blue); and subtype B in the *gp41* region (green).

A nested multiplex real-time PCR assay was developed to detect HIV-1 subtype B, CRF01_AE, CRF33_01B, and newly emerging genotypes circulating in Southeast Asia. Primers and probes of the assay targeting multiple genetic regions was designed using global reference sequences of Asian origin based on the genome organizations of the circulating subtypes and CRFs in the region. The performance of the assay was evaluated on 180 HIV-positive clinical samples (heterosexual, 31.1%; male homosexual, 22.2%; bisexual, 3.9%; injecting drug user [IDU], 10.6%; unreported risk, 32.2%). The median viral load for these samples was 6.7 log_10_ copies/mL (range: 3.0–7.0 log_10_ copies/mL). The real-time PCR results of the targeted regions (*gag p24*, *pol pro*-RT, and *env gp41* regions) were used to interpret and determine the HIV-1 subtype or CRF (representative amplification curves are shown in Figure 2

), based on the classification chart in Table 2 and the previously mentioned ΔC_q_ threshold, which was determined to be 5.6 cycles. Genotype assignment was then confirmed by direct sequencing and phylogenetic analysis. Five distinct HIV-1 genotypes were detected: subtype B (16.7%, 30 of 180), CRF01_AE (52.8%, 95 of 180), CRF33_01B (24.4%, 44 of 180), CRF53_01B (1.1%, 2 of 180), and CRF54_01B (0.6%, 1 of 180) (Table 3). Of note, real-time PCR results from eight specimens showed discordant genotype assignment when compared with nucleotide sequence analysis. These specimens were genotyped by real-time PCR as CRF01_AE (*N* = 3), CRF33_01B (*N* = 3), CRF58_01B (*N* = 1), and an “undetermined” (*p24*^+^, *pro*-RT, *gp41*^+^) genotype (*N* = 1). Recombination analysis of the *gag-pol* segments using the RIP program and bootscanning showed that these isolates were unique recombinant sequences involving CRF01_AE and subtype B, termed as CRF01_AE/B URFs (4.4%, 8 of 180). Only one specimen showed real-time PCR results (as CRF01_AE) that were not concordant with the phylogenetic inference (as CRF33_01B), as a result of the probe mismatch in the *pro*-RT region (Table 3). The panel of HIV-1 subtype B (US1, BK132, and BZ167) and CRF01_AE (CM235, CM240, and IN12) were successfully genotyped by the assay under different dilutions (10^8^ to 10^3^ copies/mL), with no evidence of cross-priming.

Overall, the accuracy of the real-time PCR assay to correctly identify an HIV-1 genotype was 95.0% (171 of 180), in which the sensitivities for subtype B, CRF01_AE, and CRF33_01B classification were 100.0% (30 of 30), 100.0% (95 of 95, 7% (43 of 44), respectively. Inter-genotype specificity was more than 95% in general, in which the specificities for subtype B, CRF01_AE, and CRF33_01B were calculated at 100.0% (150 of 150), 95.2% (79 of 83), and 97.8% (133 of 136), respectively. The assay also showed high specificity in HIV-negative specimens (100%, 50 of 50). The positive and negative predictive values of the assay for the major subtypes were both 100.0% for subtype B, 96.0% and 100.0% for CRF01_AE, and 93.5% and 99.3% for CRF33_01B, respectively. These values for other rare CRFs were not calculated as a result of inadequate sample size. The limit of detection of the assay was determined at 10^3^ copies/mL for both subtype B and CRF01_AE.

In the field test, a total of 36 subjects (8.8%) tested positive for HIV-1 infection among 410 drug injecting fishermen recruited from a rural village in Kuantan, Pahang. Real-time PCR results, in combination with sequence and phylogenetic analysis, showed the presence of HIV-1 subtype B, CRF01_AE, CRF33_01B, and CRF01_AE/B URF at 8.3% (3 of 36), 11.1% (4 of 36), 66.7% (24 of 36), and 2.8% (1 of 36), respectively. Four samples were incorrectly genotyped by the assay because of primer/probe mismatches. The overall accuracy of the assay was 86.1% (31 of 36).

## Discussion

In this study, a nested real-time PCR assay capable of detecting the common and newly emerging HIV-1 subtypes and CRFs circulating in Southeast Asia was designed and evaluated. The prevalent or endemic HIV-1 genotypes targeted by the assay were subtype B, CRF01_AE, and CRF33_01B, whereas the recently described and relatively rare recombinant lineages were CRF53_01B, CRF54_01B, and CRF58_01B. The conceptual design of the assay was based on the mosaic genome structures of the various recombinant lineages and the genetic signatures within the *p24*, *pro*-RT, and *gp41* regions that could distinguish between subtype B and CRF01_AE (Figure 1). Genotype-specific primers and probes were then designed to target either subtype B or CRF01_AE-related segments, enabling the differential detection of six HIV-1 genotypes based on the real-time PCR yield in these genetic regions (Table 2).

The newly developed genotyping assay was highly sensitive and specific, accurately determined more than 90% of the circulating HIV-1 genotypes in Kuala Lumpur, Malaysia. The results were in concordance with sequence and phylogenetic analysis for genotype determination, underlining the usefulness of the assay as an alternative method to provide rapid genotyping results, particularly in studies where the sample sizes are large. The performance of the assay was also comparable with other similar genotyping assays previously developed.^19^–^23^ It is however important to note that, in addition to the common or classical circulating genotypes, our assay is capable of detecting novel recombinant lineages (CRF53_01B, CRF54_01B, and CRF58_01B) recently reported in Malaysia.^10^,^11^,^13^ Likewise, the multi-region approach of the assay allows the detection of HIV-1 URFs in a time-effective manner,^22^ a technically established strategy for tracing the emergence of yet undefined CRF lineages in the region.

From 180 specimens collected in Kuala Lumpur between 2008 and 2012 among subjects who acquired HIV infection mainly through sexual contacts, the real-time PCR assay showed that more than half of the population were infected with CRF01_AE, followed by CRF33_01B (Table 3). The molecular epidemiological data determined by the newly developed genotyping tool was largely in concordance with previous studies that reported the predominance of HIV-1 CRF01_AE among the sexual population during the 2003–2010 period.^6^,^29^ Although the results suggest a consistent distribution of genetic diversity in the last decade, testing of recently collected samples showed the presence of novel recombinant genotypes such as CRF53_01B and CRF54_01B, albeit at low frequencies. The availability of a rapid and versatile genotyping assay will therefore facilitate the tracking of unique or complex genotypes that are otherwise difficult to detect via phylogenetic analysis based on limited HIV-1 genome coverage.

In a field test conducted among HIV-positive drug injecting fishermen from a rural village, the TaqMan assay showed high sensitivity in determining the HIV-1 genotypes. Unlike the urban population in Kuala Lumpur, HIV-1 CRF33_01B was detected at high prevalence, followed distantly by subtype B and CRF01_AE. Although both subtype B and CRF01_AE were the predominant genotypes during the early phase of the epidemic in the region,^2^ recent studies reported significant genotype replacement in which CRF33_01B became the major circulating genotype among the IDUs.^8^,^12^ The widespread dissemination of CRF33_01B among IDUs further reinforced the epidemiological and public health impact attributed to emerging recombinant lineages in the region.

The performance of the genotyping assay, however, could be affected by nucleotide mismatches in the primer and/or probe binding regions. As indicated in Table 1, the specificity of the assay is mainly dependent on single nucleotide modification near the 3′-end region of the primer. Amplification may occur in samples where mismatches were present in both the forward and reverse primers, producing false positive fluorescent signals that can be detected at the late phase of the amplification cycles. In addition, similar to other multi-region PCR assay, amplification efficiency may vary between different genetic regions in the genome.^22^ Given the limited potential sites for primer design, particularly for a highly diverse virus such as the HIV-1, such a problem is difficult to avoid in assay design. Therefore, to enhance the power of differential identification for various HIV-1 subtypes and CRFs, the difference in C_q_ values between the target genes was used to assess the PCR efficiency.^25^ Another possible factor that could affect the performance of the assay is the presence of mixed infections by multiple HIV-1 genotypes. For example, if a specimen contains both subtype B and CRF01_AE, the real-time PCR assay will probably show positive detection for all three target regions (*p24*^+^, *pro*-RT^+^, *gp41*^+^), in which case the sample will be misclassified as CRF58_01B according to Table 2. Clonal sequencing and phylogenetic analysis may be required for precise genotype classification. Cases of mixed infections, however, were not apparent in this study. Finally, although the real-time PCR assay is capable of detecting unique recombinant lineages, such descriptions can only be limited to the genetic regions targeted by the assay. Inter-genotype recombination event(s) that occur outside the *p24*, *pro*-RT, and *gp41* regions could be missed by the assay and as a result, the degree of viral genetic diversity or complexity will be underestimated as a result of mis-classification. Expanding the assay coverage beyond these regions will certainly improve the resolution for URF detection,^22^ but the proportionate increase in cost and labor may restrict the use of the assay in resource-limited settings. In any case, if the cost for genetic sequencing is not the limiting factor, a genotyping method that targets more genetic regions or the complete viral genome will be useful for accurate and reliable genotype classification.

In summary, the newly developed genotyping assay is accurate, simple to perform, and suitable for genotype determination in areas where the genetic diversity of HIV-1 is high.^30^ The availability of a sensitive real-time PCR assay that can detect a panel of circulating genotypes simultaneously may be useful for continuous molecular epidemiological surveillance, particularly in the Southeast Asia region.

## Figures and Tables

**Table 1:** Primers and probes designed for the real-time PCR HIV-1 genotyping assay

	Sequence 5′ to 3′*	HXB2 location (nt)
*p24* region		
Outer Primers		
Forward	GGTGCGAGAGCGTC	793–806
Reverse	ATGCTRTCATCATYTCTTC	1819–1837
Inner Primers		
Forward	ATGGGTRAARGTARTAGAAGAAAAGGG	1251–1277
Reverse	CTGCCTGRTGYCCYCCCACTA	1358–1378
Probe	FAM-CCCACAAGATYTAAA-MGB	1329–1343
*pro-RT* region		
Outer Primers		
Forward	CAGGAGCWGATGAYACAGT	2329–2347
Reverse	AATAYTGGRGTATTRTATGGA	2711–2731
Inner Primers		
Forward	YCAGMTTGGNTGYACTTTAAATTTYCCC	2525–2552
Reverse	TTTYCCTTCYTTYTCCATTTCKG	2665–2687
Probe	VIC-ACARTGGCCATTRACAGA-MGB	2615–2632
*gp41* region		
Outer Primers		
Forward	TGTTGCAACTCACAGTCT	7918–7935
Reverse	TGARTATCCCTKCCTAAC	8346–8363
Inner Primers		
Forward	TGGGGNTGYTCTGGAAARC	8010–8028
Reverse	AYYAAGCCTCCTACTAYYATTATGAA	8277–8302
Forward	TGGGGHTGCTCTGGAARAC	8010–8028
Reverse	AYYAARCCTCCTAYTAYCATTATGAA	8277–8302
Probe	NED-CARCARGAAAWRAATGAA-MGB	8178–8195
Alternative probe	NED-TGGGANARAGAAATT-MGB	8115–8129

* Underlined characters in the inner primers indicate signature nucleotide positions that can discriminate between human immunodeficiency virus type 1 (HIV-1) subtype B and CRF01_AE.

**Table 2:** Classification of HIV-1 genotypes based on the real-time PCR amplification and detection of the *p24*, *pro*-RT, and *gp41* genes

HIV-1 genotype	*p24* (CRF01_AE-specific)	Primers and probes	*gp41* (subtype B-specific)
		*pro-*RT (subtype B-specific)	
Subtype B	**−**	+	+
CRF01_AE	+	**−**	**−**
CRF33_01B	+	+	**−**
CRF53_01B*	+	**−**	**−**
CRF54_01B	**−**	+	**−**
CRF58_01B	+	+	+

* CRF01_AE and CRF53_01B shared similar real-time PCR yield.

“+” and “–” signs indicate positive and negative real-time PCR detection, respectively.

HIV-1 = human immunodeficiency virus type 1.

**Table 3:** HIV-1 genotype determination by real-time PCR assay and phylogenetic analysis

HIV-1 genotype by phylogenetic analysis	HIV-1 genotype by real-time PCR						
	Subtype B	CRF01_AE / CRF53_01B	CRF33_01B	CRF54_01B	CRF58_01B	Undetermined	Total
Subtype B	30						30
CRF01_AE		95					95
CRF33_01B		1	43				44
CRF53_01B		2					2
CRF54_01B				1			1
CRF58_01B							0
CRF01_AE/B URF		3	3		1	1	8
Total	30	101	46	1	1	1	180

HIV-1 = human immunodeficiency virus type 1; URF = unique recombinant form.
